# Oral Health Status and Practices, and Anthropometric Measurements of Preschool Children: Protocol for a Multi-African Country Survey

**DOI:** 10.2196/33552

**Published:** 2022-04-27

**Authors:** Maha El Tantawi, Morenike O Folayan, Ahmed Bhayat

**Affiliations:** 1 Department of Pediatric Dentistry and Dental Public Health Faculty of Dentistry, Alexandria University Alexandria Egypt; 2 Department of Child Dental Health Faculty of Dentistry Obafemi Awolowo University Ile-Ife Nigeria; 3 Department of Community Dentistry School of Dentistry University of Pretoria Pretoria South Africa

**Keywords:** oral health, early childhood caries, oral hygiene, dietary intake, Africa, preschool children, dentistry, oral disease

## Abstract

**Background:**

Oral diseases are among the most prevalent conditions with significant impact on the growth and development of young children. Data are required to plan effectively for the management of early childhood caries (ECC) and other oral diseases in this age. There are currently very few African countries with updated and nationally representative data on ECC prevalence, and risk indicators and regional data on ECC and other oral diseases are scarce.

**Objective:**

We aim to determine the oral health status and practices, dietary intake, and anthropometric measurements of preschool children in several African countries.

**Methods:**

A cross-sectional study will be conducted in several African countries using a standardized questionnaire and clinical examination for data collection from healthy preschool children in kindergartens and primary health care facilities. The clinical examination will assess ECC using the decayed, missing due to caries, and filled teeth (dmft) index according to the World Health Organization (WHO) criteria, dental erosion (using the Basic Erosive Wear Examination Index), deciduous molar hypomineralization (using the European Association of Paediatric Dentistry criteria), dental fluorosis (using Dean’s Index), oral hygiene status (using the Oral Hygiene Index Simplified), and oral mucosal lesions. Oral hygiene habits and dental visits will be assessed using the WHO child questionnaire, and dietary intake will be assessed using the Food and Agriculture Organization method. Anthropometric measurements will be obtained following the International Society for the Advancement of Kinanthropometry standard protocol, and the children’s nutritional status will be assessed following the WHO child growth standards. To train and calibrate examiners, educational resources and electronic forms will be used to reach interexaminer and intraexaminer reliability with κ>0.6. Descriptive analysis will determine the prevalence of clinical conditions by age and sex. Bivariate analysis and multivariable regression will assess associations between the clinical conditions and sociodemographic factors, and oral health behaviors.

**Results:**

Data collection will begin after approvals and ethical clearance are obtained. The first stage will include 3 countries, namely Egypt, Nigeria, and South Africa, and collaborators from other African countries will join afterward.

**Conclusions:**

This study will lay down the foundations for using validated tools to collect data on the oral health of young children in Africa, allowing researchers from different countries across Africa to collect standardized data on ECC and other oral conditions. This will facilitate comparisons and analysis of risk factors that might be unique to the African continent. The results will provide baseline data on the prevalence of oral diseases and enable planning to address the treatment needs of young African children and design programs to prevent oral diseases in the African continent.

**International Registered Report Identifier (IRRID):**

PRR1-10.2196/33552

## Introduction

Oral diseases and dental caries are public health problems [1,2]. Early childhood caries (ECC), caries in children younger than 6 years of age, is one of the most common childhood diseases globally affecting up to 70% of children from disadvantaged communities and developed countries [3,4]. In addition, 8% of children worldwide are affected by untreated ECC [5].

Most African countries have no or outdated ECC data. According to available data, ECC prevalence ranges from 14.9% in Nigeria to 86% in Gambia [6]. Caries is multifactorial with many factors implicated including bacteria, poor diet (such as excessive consumption of refined carbohydrates), and poor oral hygiene [7].

The consequences of ECC include pain, loss of appetite, loss of school days, and poor quality of life [3,8]. Children with ECC are more likely to have caries in their adult lives, and hence, prevention of caries is essential for the well-being of children [3,4]. Data on the presence and severity of ECC have been mainly reported using the decayed, missing due to carries, and filled teeth (dmft) index of the World Health Organization (WHO). However, this index does not report on the clinical consequences of ECC [9]. The pufa index addresses this gap by assessing visible extension of caries to pulp (p), ulcerations of the oral mucosa due to root fragments (u), fistulae (f), and abscesses (a) [9].

In addition to caries, children’s hard dental tissues may be affected by other diseases such as dental erosion (DE) and developmental defects of enamel (DDE) such as fluorosis and deciduous molar hypomineralization (DMH). DE is caused by extrinsic (ingestion of acidic food, drinks, and acidic medications) and intrinsic (regurgitation of gastric contents into the mouth through gastroesophageal reflux) factors [10]. DE affects the enamel, dentine, and even pulp resulting in pain, sensitivity, and discomfort [10]. Its prevalence is increasing [10] with current prevalence among young children ranging between 20% to 70% [11].

DDE such as fluorosis, enamel hypoplasia, and DMH are childhood oral lesions. Dean’s Index has been used to measure fluorosis across the globe and is recommended by the WHO [12]. Approximately 12% of children aged 4 to 5 years have fluorosis in 1 or more teeth [13]. Data from Africa about DMH are sparse with little information about the relevant risk factors, and this limits the design and implementation of risk prevention programs for these lesions.

Diet is a risk factor for ECC and DMH [14]. Night-time bottle feeding, intake of sweetened juice, nocturnal breastfeeding after 12 months of age, and prolonged use of a pacifier covered in sweetened substances increase the risk of developing ECC [14]. Dietary diversity measured by the number of consumed food groups over a specified period is an indicator of diet quality, micronutrient adequacy, and micronutrient density [15]. The nutritional status of children can be assessed by anthropometry [16] including height-for-age, weight-for-age, weight-for-length, and weight-for-height z-scores and the mid-upper arm circumference [16-18].

Data about oral diseases, oral health practices, and dietary intake in young children in Africa are scarce. A situational analysis is needed to shed light on these conditions in different African countries to identify oral health needs and help plan oral health services based on evidence in addition to the provision of advice about dietary and oral hygiene practices. The aim of the study is to assess the prevalence of ECC and its severity using the pufa index, DDE, DMH, DE, oral health practices including oral hygiene habits, and the diversity of dietary intake in addition to anthropometric measurements of preschool children in several African countries.

## Methods

### Design, Setting, and Participants

A cross-sectional study will be used to collect data from children aged 1 to 5 years, who are free from diagnosed physical or intellectual disabilities and whose parents or guardians consent to join the study. These children will be recruited from wellness centers, kindergartens, early childhood centers, vaccination sites, and other primary health care facilities depending on the country-level context.

### Sample Size

The required sample size can be determined [19] using a 5% margin of error, 95% CI, and the estimates of prevalence of oral diseases based on the literature with accommodation of design effects because of stratification and clustering of the sample [20], as shown in Table 1.

The maximum calculated sample size is 766 and with 20% expected nonresponse, the required sample size ranges from 334 at 10% prevalence to 920 at 50% prevalence. Researchers in each country or location will adapt these estimates based on which oral health outcomes they are studying and the previous estimates of prevalence at their site. In the absence of previous estimates, the working prevalence for sample size estimation would be 50%.

**Table 1:** Estimates of prevalence of oral diseases and required sample size.

Oral disease prevalence (%)	Sample size based on simple random selection	Sample size considering design effects because of clustering and stratification
10	139	278
20	246	492
30	322	644
40	368	736
50	383	766
60	368	736
70	322	644
80	246	492
90	139	278

### Sampling Technique

Multistage stratified cluster sampling will be used, including stratification by the area of residence (rural and urban) according to the population distribution of each country and by age into the following five age groups: 12-23 months, 24-35 months, 36-47 months, 48-59 months, and 60-71 months. All participants in a facility or at a site (cluster) will be included. Participants and facilities will be randomly selected per stratum.

### Data Collection Tools

#### Clinical Examination

Plaque will be recorded using the Simplified Oral Hygiene Index as described by Green [21]. The following teeth and surfaces will be included: 55 buccal surface, 51 labial surface, 65 buccal surface, 75 lingual surface, 71 labial surface, and 85 lingual surface. Each tooth will be scored as follows: 0-no plaque; 1-plaque less than a third of the tooth surface; 2-plaque more than a third but less than two-thirds; and 3-plaque covering more than two-thirds of the tooth surface. The average individual score will be calculated by adding the scores of each tooth and dividing by 6, which is the number of teeth.

ECC examination will be performed under natural or fluorescent lighting with the participant sitting on a chair according to the WHO method of examination [12]. The examination is visual-tactile, conducted through visual observation and using a blunt probe such as the ball-ended WHO probe or a periodontal probe. When in doubt, examiners should assign lesser scores. Tooth surfaces should not be routinely probed. The probe should be used only to confirm possible enamel breakdown. The dmft index will be used to record the ECC status in primary teeth. Only teeth with cavitated caries will be diagnosed as decayed, whereas those with early white spot lesions or fissure sealants will be recorded as sound. The mesial and distal surfaces with tight contacts and no obvious signs of ECC lesions shall be coded as sound. Code x is to be used for a tooth that cannot be seen because it is unerupted, when a primary tooth has exfoliated, or when a permanent incisor is about to erupt or has already erupted. An examination form will be used to capture the clinical findings (see Multimedia Appendix 1).

The pufa index will be used to assess the severity of dental caries [9]. Decayed teeth will be classified as “p” if pulp is visible, “u” if there is ulceration of the oral mucosa due to root fragments, “f” if there is a fistula, and “a” if an abscess is present. Soft tissue lesions from the surrounding tissue that are not related to a tooth or ECC will not be recorded. Only 1 score will be assigned per decayed tooth.

The prevalence and severity of DE will be measured using the Basic Erosive Wear Examination Index [22]. The buccal, occlusal, and palatal surfaces will be examined, and the highest score for each sextant will be recorded. The sum of the scores from each sextant will be added to obtain the individual score. The sextants will be classified as follows: Sextant 1: tooth #54-55, Sextant 2: #53-63, Sextant 3: #64-65, Sextant 4: #74-75, Sextant 5: #73-83, and Sextant 6: #84-85.

To assess DMH, teeth will be examined wet after debris removal using a piece of gauze. Each surface of all primary teeth will be screened for the following based on the European Association of Paediatric Dentistry’s diagnostic criteria for (DMH): score 1-absence of demarcated opacities, score 2-posteruptive enamel breakdown, score 3-atypical restoration reflecting the distribution of hypoplastic enamel, and score 4-extracted teeth owing to DMH [23].

Dental fluorosis will be recorded using Dean’s Index as recommended by the WHO [12,24]. All primary teeth will be included in the examination. The scoring includes normal: smooth, glossy, pale creamy-white translucent enamel surface; questionable: a few white flecks or white spots; very mild: small opaque, paper- white areas covering less than 25% of the tooth surface; mild: opaque white areas covering less than 50% of the tooth surface; moderate: all tooth surfaces affected, marked wear on biting surfaces, and with possible brown staining; and severe: all tooth surfaces affected, discrete or confluent pitting, and brown stain present.

Oral lesions not associated with dental caries will be identified and recorded according to the WHO classification [25], including leukoplakia, lichen planus, necrotizing ulcerative gingivitis, and candidiasis in addition to others. The type and location of the condition will be recorded.

To obtain an acceptable level of data reliability for ECC, DMH, and DE among examiners, a reference calibrating examiner (gold standard) in each country will be selected, who is a dentist with a record of publishing studies on ECC, DDE, or erosion in preschool children. The gold standard examiners will later calibrate the examiners in their respective countries. Training of the gold standard examiners will be conducted using photographs of lesions followed by an internet-based quiz asking examiners to assign diagnostic codes that will be compared to predefined diagnoses set by consensus within the core study team (authors of this paper: MET, MOF, and AB). The technique of using photographs for training and calibration has been used previously and has proved valid [21]. In addition, this method is also recommended by the WHO [12]. Once trained using the photographs, the gold standard examiners will conduct a field-based calibration exercise for the other examiners comparing the examiners’ diagnostic codes to those of the gold standard per country. Reliability will be assessed between a group of examiners (interexaminer reliability) and between the same examiner at different time points (intraexaminer reliability). This is done by comparing the results of examining the teeth of 5 or more children among examiners and duplicates of the examination of the same children, respectively. Discrepancies are resolved by discussion and the exercise will be repeated until reaching at least 90% agreement with κ>0.6, indicating substantial to perfect agreement [26]. The Bangdiwala (B) statistic may be a suitable alternative to use in case of low prevalence of oral health outcomes [27].

#### Questionnaire

A standardized questionnaire (see Multimedia Appendix 2) will be used to collect data on oral hygiene habits [12] and dietary intake [28]. The questionnaire consists of the following sections:

Section 1 assesses the sociodemographic background including the children’s age, sex, and familial factors such as parental education and occupation, as well as the children’s rank, weight at birth, and history of breastfeeding.

Section 2 pertains to the history of several medical conditions and childhood illnesses. These include the history of medical conditions that may predispose children to malnutrition, enamel hypoplasia, and an increased risk of prolonged use of sweetened medication.

Section 3 assesses caries risk behaviors. These include the frequency of eating refined carbohydrates between meals, oral hygiene habits, history of dental visits, and parental assessment of child health based on the WHO questionnaire [12].

Section 4 is a 3-day food diary [29]. The diary takes notes of the food consumed at mealtimes and in between meals, including a weekend. Parents are expected to write down everything the child eats and drinks including how much was eaten using an estimate of portion sizes.

Section 5 is the Minimum Dietary Diversity Questionnaire assessing whether the child had eaten any of the 15 food groups in the last 24 hours [28]. Section 6 assesses the frequency and quantity of intake of 12 categories of sugary foods and oils [30].

The questionnaire will be completed before the oral examination is conducted and after written consent for participation in the study is obtained. The questionnaire can be translated into local languages and pilot tested to ensure accuracy and cultural appropriateness.

#### Anthropometric Measurements

Anthropometric measurements will be taken according to the standard guidelines [16,17]. Nutritional status will be determined using the WHO AnthroPlus software, which contains the WHO child growth standard for children aged 0 to 5 years [31]. Data on height and weight will be collected in line with the standard protocol of the International Society for the Advancement of Kinanthropometry [32]. Children will remove shoes and any heavy clothes before having their height and weight measured. Height will be measured to the nearest 0.1 cm with a portable stadiometer (Seca 217) and weight will be measured to the nearest 0.1 kg using a portable digital scale with remote display (generic electronic digital weighing scale) and the weighing scale will be zero-balanced before each child steps onto it. Measurements will be recorded after the fluctuations on the digital screen stop. Records will be taken after 2 consistent readings are obtained.

### Ethical Considerations

Approval for the study will be obtained from the ethics and research committee of each country. Consent will be sought from caregivers for their participation and the participation of their children in the study. Where culturally appropriate, permission for the mother’s participation in the study and consent for child’s participation will be sought from the husband or father. The child’s caregiver will be provided with the researchers’ names and affiliations, detailed contact information of the principal investigator and the institutional research and ethics committee, study title, objectives, methods, risks, and benefits. Participants will also be informed that they have the right to withdraw from the study and will be provided with explanations regarding how the confidentiality of the provided information will be ensured. The signed consent form will be duplicated. One of the copies will be given back to the mother, whereas the other will remain with the principal investigator. Only those who give their consent to participate in the study will be enrolled.

At the end of the study, participating caregivers shall be given an information sheet that provides details on the causes and prevention of oral diseases. Participating children will receive oral hygiene instructions once the screening is completed. Parents or legal guardians will be notified of their child’s oral health status, anthropometric status, and dietary intake diversity. If treatment is required, a referral letter will be given to them.

### Statistical Analysis

#### Data Management

Data will be entered directly into an electronic form constructed using SurveyMonkey (Momentive Inc). Alternatively, a recorder may enter data on paper forms and later transfer them to the database. The examination form will show that the surfaces of each tooth are arranged in the same order for all teeth on the right and left sides of the mouth, namely occlusal, mesial, distal, buccal, and lingual, rather than the usual mirror image system displayed on dental charts. This arrangement is designed to make it easier for recorders to enter data in a systematic manner. The master database will be backed up each day and converted to PDF. However, data will be maintained as Excel (Microsoft Corporation) files for analysis. Deidentification of the records will be done by removing any personal identifiers that can be used to trace the data to specific participants.

#### Data Analysis

Data will be imported into SPSS (IBM Corporation) for analysis. Descriptive analysis will be conducted to determine the prevalence of clinical conditions by age and sex. Bivariate analysis will be conducted to test associations between the presence of clinical conditions and sociodemographic factors as well as oral health habits. Anthropometric data will be analyzed using the WHO AnthroPlus software [16]. Dietary diversity scores will be calculated. Minimum dietary diversity will be included when children complete the survey on 5 or more out of 7 possible food groups.

Multivariable regression will be used to determine the associations controlling for confounders with calculation of regression estimates and their 95% CIs. The goodness-of-fit statistics of the models will be calculated. Significance will be set at 5%.

## Results

This study is not funded and principal investigators at each site or in each country are expected to secure the funds needed to conduct the study. Egypt, Nigeria, and South Africa will participate in the first stage of data collection with more countries to be invited to join in the next stage(s). Participant recruitment is expected to begin once ethical clearance is obtained in the respective countries.

## Discussion

### Study Objectives and Outcomes

This study will provide information about the oral health of preschool children in African countries where oral health data are scarce. Several mother and child health programs are instituted in many African countries through governmental efforts in addition to developmental assistance for health. Generating evidence about the oral health needs of young children can support the integration of oral care for children into these existing programs.

It is expected that the prevalence of dental caries will be high in most of the countries in Africa, especially those with transitional economies. The improved financial ability of citizens increases access to high-carbohydrate diets. However, health system development may be slower than the rate of economic growth, thereby reducing the possibility of providing preventive and curative care to children. Macrolevel factors are important in understanding the risk of children to oral diseases including ECC. Country context is needed when reflecting on the prevalence of ECC and the proposals for ECC-related policies and guidance.

Household risk factors for oral diseases and ECC are also important when discussing the study findings. Socioeconomic status may increase the risks related to nutrition, dental service use, neighborhood, and residential location in addition to risks associated with knowledge, attitude, and practice. In addition, cultural practices are critical factors that may affect dietary practices of infants and children. Discussions about the prevalence of ECC will take this into cognizance.

At the individual level, each child may have risk factors for oral diseases and ECC including defective dental enamel (enamel hypoplasia, DMH, and fluorosis). Collecting data on dietary intake and obtaining anthropometric measurements help assess the nutritional status of each child and identify how nutritional factors may be a risk factor for oral diseases. This is especially important for Africa where malnutrition is still a major risk factor for under-5 mortality and morbidity. The prevalence levels are 5.3%, 30.7%, and 6% for overweight, stunting, and wasting, respectively [33]. Understanding the relationships between malnutrition and oral health diseases will facilitate the development of integrated health programs that can address the oral and general health of children [34]. Such integration will likely occur at minimal costs, as it will likely fit into the targets of many existing country programs that address malnutrition as a developmental problem.

The study also aims to systematically collect data using standardized forms and methods. The developed calibration methods can be used to support data collection in future projects. The study would be the first to provide comparisons across African countries in the field of oral health, especially in young children. In addition, the study will help many countries in Africa to provide more recent data on young children’s oral health and ECC. The findings would be generalizable to entire countries because the used sampling strategy ensures representation of different regions and subgroups.

The study findings will be disseminated through the publication of the first regional profile of ECC, focusing on the prevalence of ECC in Africa. This will provide evidence to support or dispel assertions about the high prevalence of ECC in the region. Moreover, countries will be able to publish and disseminate country-specific data generated using the methods described in this protocol.

The study may have some limitations. First, there may be language barriers when answering the dietary survey. This can be addressed by translating the survey into different languages where required. The survey tool also includes photographs of food items to help parents understand the meaning of the used terms. Country-level researchers can change these photographs and replace them with those of similar local items. Second, there may be challenges in accessing rural populations due to transportation or logistic issues. In these cases, researchers can send the survey forms beforehand to be collected on the day of the examination, when the anthropometric assessments will be done. Third, the cross-sectional design of the study is suitable for exploring population parameters and program planning but cannot support causality, and longitudinal studies would be needed to overcome this limitation. Fourth, data collection about oral hygiene habits and dietary intake is subject to recall and social desirability biases, as is the case with all studies using questionnaires to elicit information. The use of standardized and validated tools such as those used by us reduces these biases. Fifth, recruiting nationally representative samples may be difficult and external funds would be needed to help cover the associated expenses. Furthermore, focusing on selected indicators of oral health outcomes and practices may help tailor the protocol to fit available resources in different countries.

### Conclusions

The methods described in this protocol provide guidance for researchers in Africa to gather data on oral diseases and anthropometric scores in young children using a standardized and validated data collection tool. The data can help generate descriptive information on prevalence and risk indicators for oral health diseases in preschool children in the region.

## Appendix

Multimedia Appendix 1 Clinical examination form.

Multimedia Appendix 2 Questionnaire.

## Abbreviations

DDE: developmental defects of enamel
DE: dental erosion
dmft: decayed, missing due to caries, and filled teeth
DMH: deciduous molar hypomineralization
ECC: early childhood caries
pufa: pulp, ulcerations of the oral mucosa due to root fragments, fistulae, and abscesses
WHO: World Health Organization
