# Supplementary material for: Oral Health Status and Practices, and Anthropometric Measurements of Preschool Children: Protocol for a Multi-African Country Survey
Source: JMIR Res Protoc. 2022 Apr 27;11(4):e33552. doi: 10.2196/33552 (PMC9096655; doi:10.2196/33552)
Supplement: Multimedia Appendix 1 [file resprot_v11i4e33552_app1.docx]

Multimedia Appendix 1 for *Oral health status, and practices and anthropometric measurements of preschool children: protocol for a multi-African country survey*

**Clinical Examination Form**

**Date: Examiner name:**

**Child name: Child code:**

**Section 1: ECC**

dmft index: pufa score:

|  | 55 | 54 | 53 | 52 | 51 | 61 | 62 | 63 | 64 | 65 |
| --- | --- | --- | --- | --- | --- | --- | --- | --- | --- | --- |
| dmft score |  |  |  |  |  |  |  |  |  |  |
| pufa score |  |  |  |  |  |  |  |  |  |  |
|  | 85 | 84 | 83 | 82 | 81 | 71 | 72 | 73 | 74 | 75 |
| pufa score |  |  |  |  |  |  |  |  |  |  |
| dmft score |  |  |  |  |  |  |  |  |  |  |

d= caries m= missing f= filled

p=pulpal involvement u=ulceration (gingiva) f=fistula a= abscess

**Treatment required_____________________________________________**

**Section 2: Dental Erosion, DMH and Fluorosis**

|  | 55 | 54 | 53 | 52 | 51 | 61 | 62 | 63 | 64 | 65 |
| --- | --- | --- | --- | --- | --- | --- | --- | --- | --- | --- |
| Fluorosis |  |  |  |  |  |  |  |  |  |  |
| DMH |  |  |  |  |  |  |  |  |  |  |
| Erosion |  |  |  |  |  |  |  |  |  |  |
| Erosion |  |  |  |  |  |  |  |  |  |  |
| DMH |  |  |  |  |  |  |  |  |  |  |
| Fluorosis |  |  |  |  |  |  |  |  |  |  |
|  | 85 | 84 | 83 | 82 | 81 | 71 | 72 | 73 | 74 | 75 |

**Dental Erosion** **Dental fluorosis**

Severity: Status:

Number of teeth affected: Number of teeth affected:

0= No sign of erosion normal mild

1= Enamel erosion questionable moderate

2=Dentinal erosion very mild severe

3= Pulp involvement

**Section 3: Oral Mucosal Lesions**

Condition/s: ______________________ Location/s: _____________________

0= No abnormal condition 0=Vermillion border

1=Tumour 1=Commissures

2=Leukoplakia 2=Lips

3=Lichen planus 3= Sulci

4=Ulceration 4= Buccal mucosa

5=Necrotising Ulcerative Gingivitis 5= Floor of mouth

6=Candidiasis 6= Tongue

7=Abscess 7= Hard and /or soft Palate

8=Other condition (specify if possible) 8= Alveolar ridge/gingiva

**Section 4: Simplified Oral Hygiene Index**

Debris Index

|  | 55 Buccal | 51 Labial | 65 Buccal | 75 Lingual | 71 Labial | 85 Lingual | Total score |
| --- | --- | --- | --- | --- | --- | --- | --- |
| Score |  |  |  |  |  |  |  |

0- no debris

1-less than 1/3

2- More than a third and less than 2/3

3- More than 2/3
