# Supplementary material for: Oral Health Status and Practices, and Anthropometric Measurements of Preschool Children: Protocol for a Multi-African Country Survey
Source: JMIR Res Protoc. 2022 Apr 27;11(4):e33552. doi: 10.2196/33552 (PMC9096655; doi:10.2196/33552)
Supplement: Multimedia Appendix 2 [file resprot_v11i4e33552_app2.docx]

Multimedia Appendix 2 for *Oral health status, and practices and anthropometric measurements of preschool children: protocol for a multi-African country survey*

**Questionnaire**

**Date: Interviewer name:**

**Child name: Child code:**

SECTION 1: Sociodemographic factors

| No | Section A1: Personal background | | | Response | |
| --- | --- | --- | --- | --- | --- |
| 1 | Age (as at last birthday) in years | | |  | |
|  | DOB | | | …./ …./ ….. | |
| 2 | Gender | | | Male ( ) Female ( ) | |
| 3 | Name of School | | |  | |
| 4 | Class | | |  | |
|  | Section A 2: Family background | | |  | |
| 5 | Residence | | | Urban ( )  Rural ( ) | |
| 6 | Birth Position (1st born, 2^nd^ born, 3^rd^ born…) | | |  | |
| 7 | Does your child/guardian live with one parent, both parents and a guardian? | | | 1. Both parents ( )  2. Single parent ( )  3. Guardian ( ) | |
| 8 | Father’s occupation | | | Professional ( )  Non professional ( )  Retired ( )  Not working ( ) | |
| 9 | Mother’s occupation | | | Professional ( )  Non professional ( )  Retired ( )  Not working ( ) | |
| 10 | Father’s level of education | 1. No formal education ( ) 4. Completed tertiary education ( )  2. Completed Primary School ( )  3. Completed Secondary School ( ) | | | |
| 11 | Mother’s level of education | 1. No formal education ( ) 4.Completed tertiary education ( )  2. Completed Primary School ( )  3. Completed Secondary School ( ) | | | |
| 12 | Did you breastfeed your child? | | Yes ( ) No ( )  If yes, for how many months did you breastfeed your child?  ( ) Less than 6 months ( ) 7-12 months ( ) longer than 12 months | | |
| 13 | What was the weight of your child at birth in Kilograms (Kg)? | | | |  |

Section 2: Medical History

| 1 | Does your child have any of the following conditions? |
| --- | --- |
| 2 | Asthma ( ) Yes ( ) No |
| 3 | Epilepsy ( ) Yes ( ) No |
| 4 | Heart conditions ( ) Yes ( ) No |
| 5 | HIV infection ( ) Yes ( ) No |
| 6 | Breathing problems ( ) Yes ( ) No |
| 7 | Kidney Problems ( ) Yes ( ) No |
| 8 | Liver problems ( ) Yes ( ) No |
| 9 | Stomach problems ( ) Yes ( ) No |
| 10 | Others (Please list) |

SECTION 3: Dental habits and oral health perception

| No | Questions | Response |
| --- | --- | --- |
|  | B1: Sugar consumption |  |
| 1 | How often does your child eat sugar containing snacks or drinks between the main meals? | 1. About 3 times a day or more ( )  2. About twice a day ( )  3. About once a day ( )  4. Occasionally; not every day ( )  5. Rarely or never eat between meals ( ) |
|  | B2: Oral hygiene |  |
| 2 a | How do you clean your child’s teeth? (multiple selections allowed) | 1. Toothbrush ( )  2. Wooden toothpicks ( )  3. Plastic toothpicks ( )  4. Thread (dental floss) ( )  5. Charcoal ( )  6. Chewstick/miswaak  7. Other (specify) |
| 2 b | How often do you or your child brush his/her teeth? | 1. Irregularly or never ( )  2. Several times a month’s (2-3)  3. Once a week ( )  4. A few (2-6) times a week ( )  5. Once a day ( )  6. Twice or more a day ( ) |
| 3 | How often do you clean in between the teeth of your child, use dental floss? | 1. Never ( )  2. Occasionally ( )  3. A few (2-3) times a week ( )  4. Once in a day ( )  5. More than one time a day ( ) |
| 4 | How often does your child use toothpaste? | 1. Always ( )  2. Quite often ( )  3. Seldom ( )  4. Not at all ( ) |
| 5 | Do you use toothpaste that contains fluoride? | Yes  No  Do not know |
|  | B3: Dental visits |  |
| 6 | When last did you take your child for a dental check-up? | 1. Within the last 6 months ( )  2. Between 6 months and 1 year ( )  3. Between 1 and 2 years ( )  4. Between 2 and 5 years ( )  5. Never ( )  6. Do not remember ( ) |
|  | B4: Parental assessment of child’ s oral health |  |
| 7 | How often during the past 12 months did your child have toothache or feel discomfort due to his/ her teeth? | Often ( )  Occasionally ( )  Rarely ( )  Never ( )  Don’t know ( ) |
| 8 | Do you think that your child has dental caries? | Yes ( )  No ( )  Not sure ( ) |

SECTION 4: Three days food diary, to be completed by the patient/parent

| Time at which snack, drink, meal… was consumed | Three consecutive days, including 2 weekdays and one weekend day (eg. *Thursday, Friday & Saturday* OR *Sunday, Monday & Tuesday)* | | |
| --- | --- | --- | --- |
| Time | Day 1 | Day 2 | Day 3 |
| Example:  06h00-07h00 | Coffee with milk & 2 sugars; buttermilk rusk | Tea with honey. White bread toast with butter & cheese | Orange juice |
| 08h00-09h00 | Cornflakes with full cream milk & 3 teaspoons of sugar | 1 fruit (apple) | Oats with milk & two teaspoons of sugar |
| 06h00-07h00 |  |  |  |
| 07h00-08h00 |  |  |  |
| 08h00-09h00 |  |  |  |
| 09h00-10h00 |  |  |  |
| 10h00-11h00 |  |  |  |
| 11h00-12h00 |  |  |  |
| 12h00-13h00 |  |  |  |
| 13h00-14h00 |  |  |  |
| 14h00-15h00 |  |  |  |
| 15h00-16h00 |  |  |  |
| 16h00-17h00 |  |  |  |
| 17h00-18h00 |  |  |  |
| 18h00-19h00 |  |  |  |
| 19h00-20h00 |  |  |  |

SECTION 5- Minimum dietary diversity questionnaire

(Kindly indicate if you fed your child with any of the following groups of food in the last 24 hours)

| No. | Food groups | Examples | Yes = 1  No = 0 |
| --- | --- | --- | --- |
| 1 | CEREALS | Corn/Maize, Rice, Wheat, Sorghum, Millet or any other grains or foods made from these (e.g. bread, noodles, porridge or other grain products) |  |
| 2 | WHITE ROOTS AND TUBERS | White yam, White cassava, Cocoyam or other foods made from roots |  |
| 3 | VITAMIN A RICH  VEGETABLES | Pumpkin, carrot, squash, sweet potato + other locally available vitamin A rich vegetables (e.g. red sweet pepper) |  |
| 4 | DARK GREEN  LEAFY  VEGETABLES | Dark green leafy vegetables such as spinach |  |
| 5 | OTHER  VEGETABLES | Other vegetables (e.g. tomato, onion, eggplant) + other locally available vegetables |  |
| 6 | VITAMIN A RICH  FRUITS | Mango, pawpaw, watermelon and 100% fruit juice made from these + other locally available vitamin A rich fruits |  |
| 7 | OTHER FRUITS | Other fruits such as apple, grape, strawberry and 100% fruit juice made from these. |  |
| 8 | ORGAN MEAT | Liver, kidney, heart or other organ meats or blood-based foods |  |
| 9 | FLESH MEATS | Beef, pork, lamb, goat, chicken, duck |  |
| 10 | FISH AND  SEAFOOD | Fresh or dried fish, shellfish, crayfish |  |
| 11 | LEGUMES, NUTS  AND SEEDS | Dried beans, dried peas, lentils, nuts, seeds or foods made from these (eg. peanut butter) |  |
| 12 | MILK AND MILK  PRODUCTS | Milk, cheese, yogurt or other milk  products |  |
| 13 | OILS AND FATS | Oil, fats or butter added to food or used for cooking |  |
| 14 | SWEETS | Sugar, honey, sweetened soda or sweetened juice drinks, sugary foods such as chocolates, candies, cookies and cakes |  |
| 15 | SPICES,  CONDIMENTS,  BEVERAGES | Spices (black pepper, salt), condiments (soy sauce, hot sauce), coffee, tea. |  |

SECTION 6- FREQUENCY AND QUANTITY OF SUGARS AND OIL CONSUMPTION

(Kindly indicate the amount of each of the listed food items your child has eaten in the last 24 hours and identify which picture best quantifies the amount taken)

| No. | Food item | Frequency | Amount | Description |
| --- | --- | --- | --- | --- |
| 1 | Table sugar | Once ( )  Twice ( )  Thrice ( )  Four times ( )  More than four times ( ) | Number of teaspoons ____  Number of tablespoons___  Number of cubes ___ | 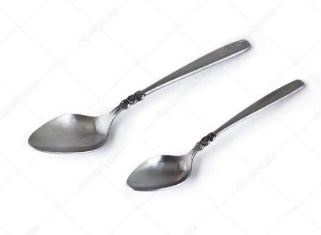 |
| 2 | Biscuits | Once ( )  Twice ( )  Thrice ( )  Four times ( )  More than four times ( ) | Number of packs  _____ | 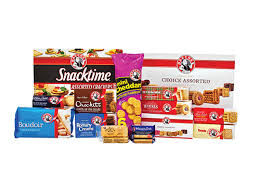 |
| 3 | Sweets | Once ( )  Twice ( )  Thrice ( )  Four times ( )  More than four times ( ) | Number of wraps of sweet  __________  Number of sticks of lollipop  ____________ | 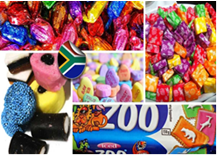 |
| 4 | Bread | Once ( )  Twice ( )  Thrice ( )  Four times ( )  More than four times ( ) | Number of slices  ___________  Number of loafs (unsliced)  ____________ | 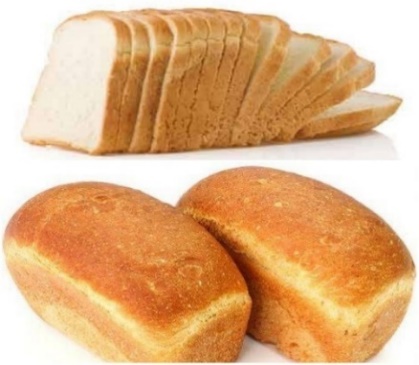 |
| 5 | Cereal such as Corn flakes. | Once ( )  Twice ( )  Thrice ( )  Four times ( )  More than four times ( ) | Amount of cups per serving  ____________ | 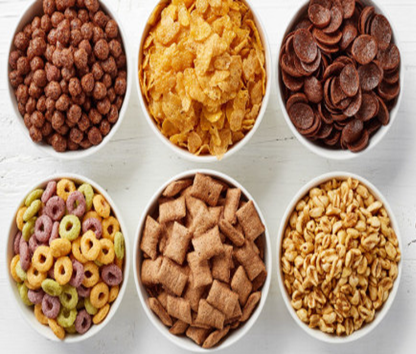 |
| 6 | Fruit juice | Once ( )  Twice ( )  Thrice ( )  Four times ( )  More than four times ( ) | Amount of cups per serving  ____________ | 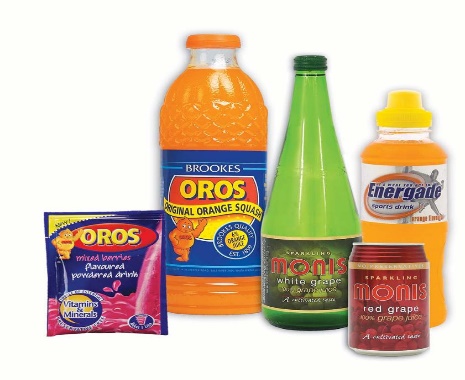 |
| 7 | Carbonated drink | Once ( )  Twice ( )  Thrice ( )  Four times ( )  More than four times ( ) | Number of bottles  ____________  Number of cans  ____________ | 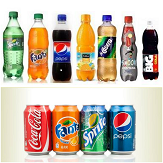 |
| 8 | Ice cream | Once ( )  Twice ( )  Thrice ( )  Four times ( )  More than four times ( ) | Number of Cups or cones or ice creams  ____________ | 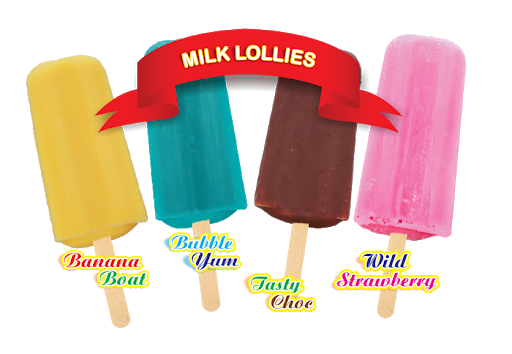 |
| 9 | Chocolate | Once ( )  Twice ( )  Thrice ( )  Four times ( )  More than four times ( ) | Number of chocolates  ____________ | 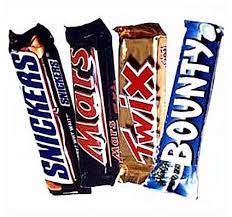 |
| 10 | Cake | Once ( )  Twice ( )  Thrice ( )  Four times ( )  More than four times ( ) | Number of cup cakes  ____________  Number of slices  ____________ | 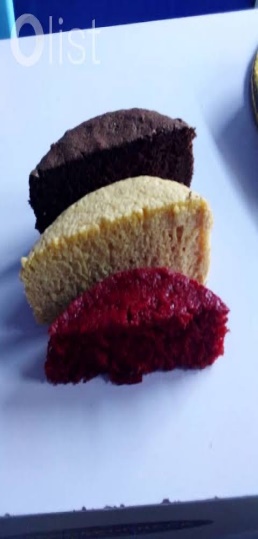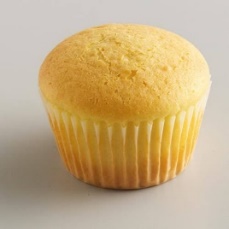 |
| 11 | Pastries – Doughnuts, vetkoek | Once ( )  Twice ( )  Thrice ( )  Four times ( )  More than four times ( ) | Number of doughnuts / vetkoek  ­­­­­­­­­­­­­____________ | 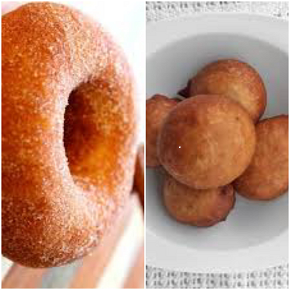 |
| 12 | Oil | Once ( )  Twice ( )  Thrice ( )  Four times ( )  More than four times ( ) | Number of tablespoons per serving  ____________ | 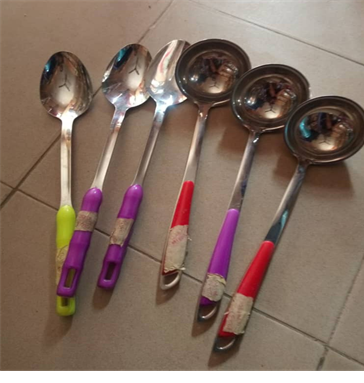 |
